# Combining a Standardized Batch Test with the Biotic Ligand Model to Predict Copper and Zinc Ecotoxicity in Soils

**DOI:** 10.1002/etc.5326

**Published:** 2022-04-18

**Authors:** Charlotta Tiberg, Erik Smolders, Mats Fröberg, Jon Petter Gustafsson, Dan Berggren Kleja

**Affiliations:** ^1^ Swedish Geotechnical Institute Linköping Sweden; ^2^ Division Soil and Water Management Catholic University of Leuven Leuven Belgium; ^3^ Department of Soil and Environment Swedish University of Agricultural Sciences Uppsala Sweden

**Keywords:** Ecological risk assessment, Biotic ligand model, Bioavailability, Soil contamination, Copper, Zinc, Batch test, Metal toxicity

## Abstract

Extraction of soil samples with dilute CaCl_2_ solution in a routinely performed batch test has potential to be used in site‐specific assessment of ecotoxicological risks at metal‐contaminated sites. Soil extracts could potentially give a measure of the concentration of bioavailable metals in the soil solution, thereby including effects of soil properties and contaminant “aging.” We explored the possibility of using a 0.001 M CaCl_2_ batch test combined with biotic ligand models (BLMs) for assessment of ecotoxicity in soils. Concentrations of Cu^2+^ and Zn^2+^ in soil extracts were linked to responses in ecotoxicity tests (microbial processes, plants, and invertebrates) previously performed on metal‐spiked soils. The batch test data for soils were obtained by spiking archived soil materials using the same protocol as in the original studies. Effective concentration values based on free metal concentrations in soil extracts were related to pH by linear regressions. Finally, field‐contaminated soils were used to validate model performance. Our results indicate a strong pH‐dependent toxicity of the free metal ions in the soil extracts, with *R* ^2^ values ranging from 0.54 to 0.93 (median 0.84), among tests and metals. Using pH‐adjusted Cu^2+^ and Zn^2+^ concentrations in soil extracts, the toxic responses in spiked soils and field‐contaminated soils were similar, indicating a potential for the calibrated models to assess toxic effects in field‐contaminated soils, accounting for differences in soil properties and effects of contaminant “aging.” Consequently, evaluation of a standardized 0.001 M CaCl_2_ batch test with a simplified BLM can provide the basis for an easy‐to‐use tool for site‐specific risk assessment of metal toxicity to soil organisms. *Environ Toxicol Chem* 2022;41:1540–1554. © 2022 The Authors. *Environmental Toxicology and Chemistry* published by Wiley Periodicals LLC on behalf of SETAC.

## Graphical abstract

A standardized batch test evaluated by a simplified biotic ligand model approach could provide a useful tool to improve site specific assessment of metal ecotoxicity in soil. EC50 = median effective concentration.

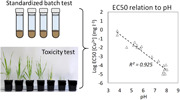

## INTRODUCTION

Soil guideline values for assessment of metal toxicity to soil organisms are usually based on total metal concentrations. However, it is well established that an assessment based directly on total concentrations often poorly predicts the metal toxicity for soil organisms on a site‐specific basis (Smolders et al., 2009). Metal bioavailability is a function of pH and other site‐specific soil properties like cation exchange capacity (CEC) and organic matter content. In addition, soil guideline values are derived from ecotoxicity tests on soils with experimental additions of metal salts, often resulting in a different response compared to similar concentrations in field‐contaminated soils (Oorts, Bronckaers, & Smolders, 2006; Smolders et al., 2004). Metals in freshly spiked soils are usually more bioavailable compared to “old” metal contaminations in field soils. Aging processes reduce bioavailability (see Lock & Janssen, 2003), while spiking with metal salt increases the ion concentration and lowers the pH (Speir et al., 1999), which generally increases metal solubility.

To refine ecotoxicity assessment based on total metal concentrations in soil, attempts have been made to establish relationships between effects in toxicity tests and soil properties. For example, Oorts, Ghesquiere, et al. (2006) found that variations in Cu toxicity to soil microbes were best explained by CEC (for nitrification), organic carbon (for substrate‐induced respiration), or soil pH (for maize mineralization). Similar results have been found for Zn, but the toxic response was also related to the background concentration of Zn (Smolders et al., 2004). The risk‐assessment tool *Threshold Calculator for Metals in Soil* (Oorts, 2020) uses toxic responses that are normalized to soil properties (e.g., CEC) based on regression relationships. In addition, *Threshold Calculator* applies so‐called laboratory‐field factors to account for differences between freshly spiked and field‐contaminated soils. Laboratory‐field factors are site‐ and element‐specific and based on empirical comparisons of toxic responses in freshly spiked and field‐contaminated or experimentally aged soils. *Threshold Calculator* uses generic laboratory‐field factors for Co, Cu, Pb, Mo, Ni, and Zn, based on data presented by Smolders et al. (2009). However, the laboratory‐field factors can vary considerably between soils, for example, for Cu and Zn approximately 1.5 orders of magnitude (Smolders et al., 2009).

Different leaching tests have frequently been used in site‐specific risk assessments to evaluate mobility and transport of contaminants. There are international standards for batch and percolation tests for testing of soil and soil‐like materials within the International Organization for Standardization (ISO) 21268 series (ISO, 2007). Leaching tests also have a potential to evaluate bioavailability to soil organisms and plants, based on the rationale that they provide a proxy for the soil solution. The soil solution is the main exposure route for soil organisms and plants, but unfortunately, it is technically challenging to sample soil solutions in sufficient quantities for subsequent analyses. Use of leaching tests with 0.001 M CaCl_2_ for evaluation of bioavailability in Tier 2 assessments is mentioned in ISO 19204 (ISO, 2017) that refers to ISO 17402, which provides guidance on selection of methods for bioavailability assessment (ISO, 2008a). Also, ISO 17402 recommends measurement of the free metal ions because “the bioavailability of complexed species is believed to be much lower than that of the free metal ion” However, further guidance on how to interpret test results is not provided in ISO 17402 or in the guidance on leaching procedures for subsequent chemical or toxicological testing of soil, ISO 18772 (ISO, 2008b).

The idea that the free metal ion is the most bioavailable species whereas the remaining metal forms, such as organically complexed species, may not be toxic was conceptualized by Morel (1983) in the free‐ion activity model. Since then, it has had a long‐standing impact on how environmental scientists conceptualize metal toxicity. However, more recently it was shown that the toxic effect related to the free metal ion alone does not give a good indication of ecotoxicity in soils. Critical effect concentrations (EC*x*) based on the free metal ion activities of Cu and Zn in soil solutions were even more variable between soils than the corresponding total soil concentrations (Smolders et al., 2009).

Poor relationships between free ion activity and ecotoxic effects have previously been observed for freshwaters, resulting in the development of the biotic ligand model (BLM) concept (Di Toro et al., 2001). In a BLM, the uptake of a metal by an organism and subsequent toxic response is the net result of chemical reactions in the solution and competition between cations on the so‐called biotic ligand, which is a receptor on the organism to which the metals can bind. Such models of different complexity have been developed. The “classical” BLM approach (Di Toro et al., 2001) uses equilibrium‐type uptake reactions for Cu^2+^ and competing ions on the biotic ligand. For algae, however, it was found that competing ions other than H^+^ did not affect Cu^2+^ toxicity significantly (De Schamphelaere et al., 2003); hence, a simpler BLM approach was proposed, focusing only on the relationship between pH and log{Cu^2+^} at a given toxicity endpoint such as the no‐observed‐effect concentration (NOEC) or the 10% EC (EC10). When available, aquatic BLMs are now recommended for incorporation of bioavailability in environmental quality standards (EQS) within the European Union (European Commission, 2018).

Applying the BLM concept to soil systems is more complicated because, in addition to the interaction on the biotic ligand, the solid–solution partitioning of metals needs to be described. Thakali et al. (2006a) and Thakali et al. (2006b) were the first to present a terrestrial BLM (TBLM) for Cu toxicity in soils based on the “classical” BLM approach, in which H^+^, Ca^2+^, and Mg^2+^ competition on the biotic ligand was taken into account and where the partitioning and speciation of Cu were described by the Windermere humic aqueous model VI (Tipping, 1998). However, the TBLM has not yet been widely used, both because of its complexity, making it difficult to calibrate, and because it assumes all solid‐phase Cu to be “geochemically active”; that is, it does not consider aging effects. Lofts et al. (2004) suggested a simpler approach (the free ion approach) in which the biotic ligand is not explicitly considered. Instead, a simple relationship between pH and log{Me^2+^} calculated from geochemically active soil metal was used to derive “critical limits” for soils based on toxicity data from the literature. The relationships are mathematically equivalent to the aquatic “algal” BLM of De Schamphelaere et al. (2003), although toxicity response functions for different organism groups are not separated. The concept has then been further developed into the free ion effective dose (FRIED) model and tested for Cu in soil solutions (Lofts et al., 2013). Neither the TBLM nor the FRIED model has been used to evaluate metal toxicity in field‐contaminated soils, and they do not account for site‐specific metal speciation in such soils. Therefore, these models cannot be used to obtain site‐specific laboratory‐field factors. Differences between freshly spiked and field‐contaminated soils can, in principle, be accounted for by extraction with a solution containing a small amount of neutral salt, for example, CaCl_2_. Such a solution will only dissolve soluble, or reversibly adsorbed, metals and not metals occluded within minerals. Hamels et al. (2014) showed that much lower total soil Zn concentrations were required to reduce plant growth in spiked soils compared to corresponding field‐contaminated soils. In contrast, similar Zn concentrations in 0.001 M CaCl_2_ soil extracts yielded similar toxicity in both sets of soils, indicating that this test accounts for differences in bioavailability between spiked and field‐contaminated soils.

The overall objective with the present study was to explore the possibility of using a standardized batch leaching test with 0.001 M CaCl_2_, ISO 21268‐2, (ISO, 2007) in combination with a BLM approach as a tool to assess the toxicity of Cu and Zn to soil organisms. The organisms' exposure to metals was assessed by linear regression models relating toxic effects to the free metal concentration and pH of the soil extracts obtained with the batch test. The idea was to provide an easy‐to‐perform procedure for site‐specific ecological risk assessment of soils that accounts not only for the availability of the metals, which is affected by soil properties and contaminant “aging,” but also for the actual exposure to dissolved metals, which is dependent on the composition (especially pH) of the soil solution. The specific objectives were (1) to calibrate a simplified BLM for Cu and Zn ecotoxicity by combining information obtained from a standardized batch test (0.001 M CaCl_2_) and data from an existing ecotoxicity database, and (2) to evaluate the model's ability to predict toxicity in field‐contaminated soils.

## MATERIALS AND METHODS

### Strategy

**Figure 1:**
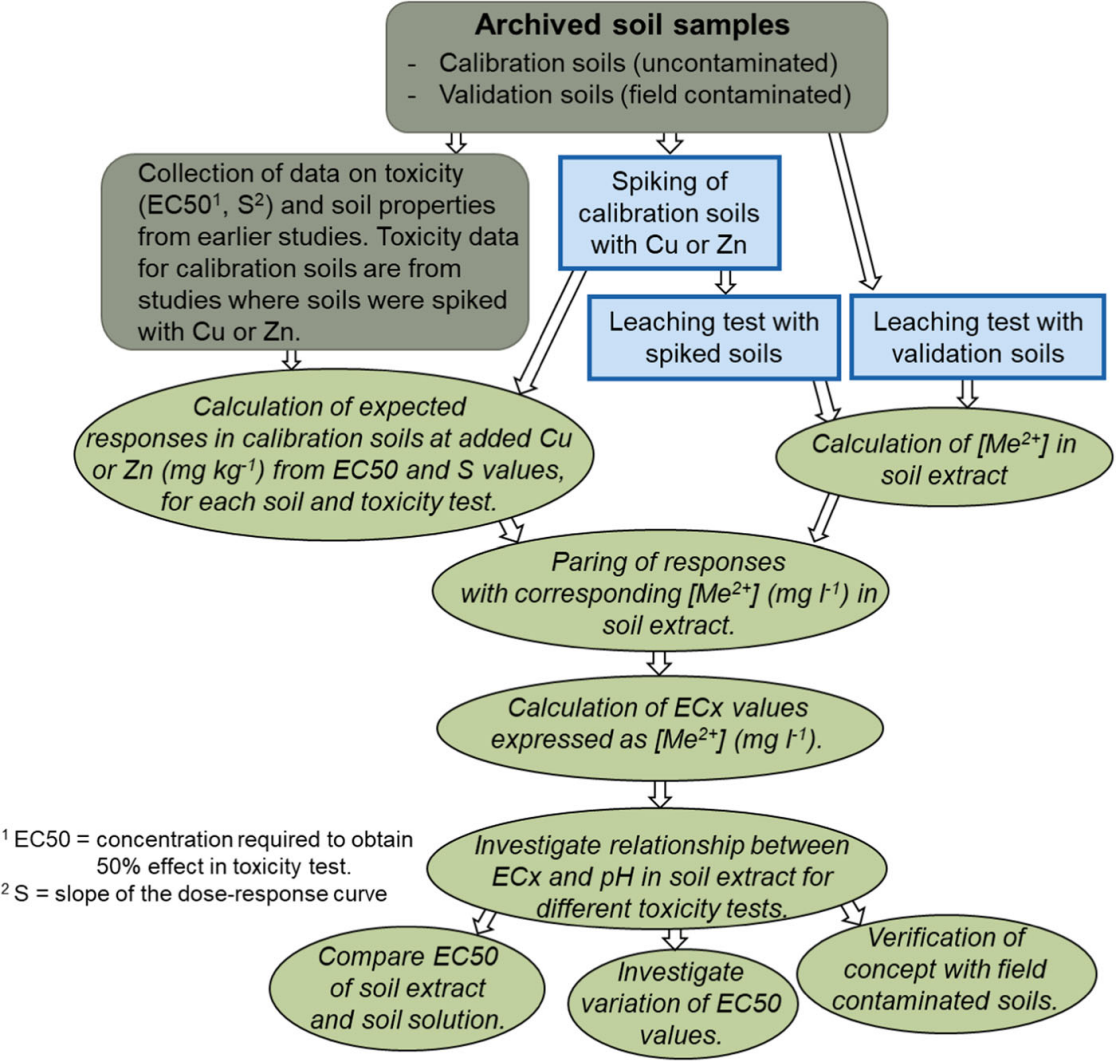
Overall strategy for calibrating and evaluating the simplified biotic ligand model based on the standardized batch leaching test (ISO 21268‐2 [ISO, 2007]). EC50 = median effective concentration; S = slope of the dose–response curve.

The overall strategy is presented in Figure 1. The idea was to use archived soils, for which dose–response relationships based on metal concentrations in soil (milligrams per kilogram dry wt) had been established for a range of endpoints, to develop a simplified BLM for assessment of metal ecotoxicity in soil. The starting assumptions of the model are those of the TBLM and the FRIED model: The free metal ion activity in the solution, not total soil concentrations, represents the toxic form; and the protons are the most important competing cations to bind on the biotic ligand. To relate the response to Cu^2+^ and Zn^2+^ activity in soil extracts (milligrams per liter) instead of the soil concentration (milligrams per kilogram dry wt), uncontaminated subsamples of the archived soils were spiked with Cu or Zn in the same way as previously done for the ecotoxicity testing. The spiked soils (“calibration soils”) were then subjected to the standardized leaching test (ISO, 2007) to produce soil extracts mimicking the soil solutions. The total and free ion concentrations of Cu and Zn in the extracts obtained from the batch tests were then linked to the responses previously determined in the various ecotoxicity tests, and EC*x* values based on metal concentrations in soil extracts were derived for each soil and endpoint. From the pH dependence of EC*x* values for each endpoint, simplified BLMs were developed. Finally, model predictions were evaluated by comparison with toxic effects in field‐contaminated soils (“validation soils”).

### Soils and toxicity tests

To develop a generic model for metal toxicity in soils, data from a set of toxicity tests performed on soils with different properties (e.g., pH, organic carbon, CEC) are needed. The tests should cover a range of soil organisms and endpoints. For this purpose, several sieved and air‐dried archived European topsoils were used (Table 1). Subsamples of the soils selected for the present study (spiked or field‐contaminated) had previously been part of studies investigating Cu or Zn toxicity to soil biota (Table 2). One set of 22 uncontaminated soils, the calibration soils, (soils 1–22 in Table 1) was used to parameterize the simplified BLM. These soils were spiked with Cu^2+^ or Zn^2+^ salts following the same procedure as in the previous ecotoxicity studies and subsequently extracted with 0.001 M CaCl_2_ in batch tests. Then, relationships were established between previously detected effects in the toxicity tests (EC*x*; milligrams per kilogram dry wt) and corresponding currently detected Cu^2+^ and Zn^2+^ in the soil extracts. Toxicity data for the spiked soils were available for up to seven tests. Another set of soils from four different sites, the validation soils (soil 23–26 in Table 1), were field‐contaminated and had been sampled along contaminant gradients/transects including an uncontaminated reference sample. Soil properties were similar along each transect, but the metal concentration varied. This set of soils was used for validation of the calibrated BLM. The uncontaminated reference sample was spiked with Cu or Zn, and all validation soils were leached with 0.001 M CaCl_2_. Ecotoxicity data (although from fewer tests) were available also for the validation soils.

**Table 1:** Properties of soils used in leaching tests

Soil no.	Soil name	Country	Soil pH^a^	Organic C^b^ (%)	CEC^c^ (cmol_c_ kg^−^ 1)	Cu^d^ (mg kg^−^ 1)	Zn^d^ (mg kg^−1^)
Calibration soils							
1	Gudow	Germany	3.0	5.12	5.8	2	7
2	Nottingham	United Kingdom	3.4	5.20	6.7	17	50
3	Houthalen	Belgium	3.4	1.86	1.9	2	8
4	Rhydtalog	United Kingdom	4.2	12.94	15.2	14	55
5	Zegveld	The Netherlands	4.7	23.32	35.3	70	191
6	Rhydtalog c.t.	United Kingdom	4.8	7.77	14.9	12	83
7	Kövlinge I	Sweden	4.8	1.63	2.4	6	21
8	Souli I	Grece	4.8	0.41	11.2	31	37
9	Kövlinge II	Sweden	5.1	2.35	4.7	8	26
10	Montpellier	France	5.2	0.76	2.5	5	16
11	De Meern	The Netherlands	5.2	10.24	29.6	55	155
12	Aluminusa	Italy	5.4	0.87	22.6	21	53
13	Zeveren	Belgium	5.7	3.48	18.9	17	76
14	Woburn	United Kingdom	6.4	4.40	23.4	22	99
15	Ter Munck	Belgium	6.8	0.98	8.9	22	54
16	Vault de Lugny	France	7.3	1.47	26.2	21	403
17	Rots	France	7.4	1.26	20.0	14	51
18	Souli II	Greece	7.4	2.61	36.3	34	51
19	Marknesse	The Netherlands	7.5	1.27	20.1	18	80
20	Barcelona	Spain	7.5	1.48	14.3	88	191
21	Brécy	France	7.5	1.51	23.5	31	251
22	Guadalajara	Spain	7.5	0.38	16.9	7	27
Validation soils							
23s	Hygum spiked	Denmark	5.4	2.1	6.7	21	38
23f	Hygum field	Denmark	5.2–5.6	2.3–3.0	8.6–10	114–825	51–60
24f	Zeveren, field	Belgium	5.6–6.2	3.2–6.1	NA	19–29	113–1409
24s	Zeveren spiked	Belgium	5.8	3.8	NA	18.9	75.9
25f	Navicello field	Italy	7.2–7.5	9.1–11.8	16–35	105–448	NA
26s	Wincheringen spiked	Germany	7.2	5.9	29	65	NA
26f	Wincheringen field	Germany	7.2–7.3	4.5–5.5	21–22	276–516	NA

^a^ Measured in 0.01 M CaCl_2_ at a soil/solution ratio of 1:5.

^b^ The difference between total carbon content was measured by ignition with a Variomax CN analyzer, and CaCO_3_ content was determined from the pressure increase after addition of HCl to closed containers including FeSO_4_ as a reducing agent.

^c^ Cation exchange capacity was measured by the silver‐thiourea method (Chhabra et al., 1975).

^d^ Boiling aqua regia extraction followed determination with inductively coupled plasma optical emission spectrometry (Perkin Elmer Optima 3300 DV).

Soils 1–22 constitute the uncontaminated calibration soils that were spiked with Cu or Zn. They are sorted from low to high pH. These soils had been spiked at different levels of Cu and Zn and were used in toxicity tests in earlier work. Soils 23–26 are field‐contaminated soils used for validation of the proposed concept. Data for calibration soils are from Smolders et al. (2004) and Oorts, Ghesquiere, et al. (2006); data for field‐contaminated soils are from Mertens et al. (2006), Oorts, Bronckaers, & Smolders (2006), and Ruyters et al. (2013).

CEC = cation exchange capacity; NA = not analyzed.

**Table 2:** Summary of toxicity data available for calibration soils

	Number of available EC50 values for calibration soils (mg kg^−^ 1 dry wt)								
Metal	PNR^a^	SIR^b^	MRM^c^	Barley root^d^	Tomato shoot^e^	Wheat shoot^f^	Springtail^g^	Earthworm^h^	No. of soils
Copper	17	16	15	16	17	–	16	14	19
Zinc	14	14	11	–	–	15	15	14	16

^a^ Potential nitrification rate (milligrams of NO_3_‐N per kilogram of fresh soil per day), nitrification at unlimited substrate (NH^4+^) by native soil organisms (ISO, 2012a).

^b^ Glucose‐induced respiration test, commonly known as the substrate‐induced respiration test, mineralization of ^14^C‐labeled glucose by native soil organisms.

^c^ Maize mineralization test, mineralization of ^14^C‐labeled maize root material.

^d^ Barley (*Hordeum vulgare*) root elongation test, based on ISO, 2012b.

^e^ Tomato (*Lycopersicon esculentum*) shoot yield test, dry matter yield of tomato shoots, based on ISO, 2012c.

^f^ Wheat (*Triticum aestivum*) shoot test, dry matter yield of wheat shoots.

^g^ Springtail (*Folsomia candida*). Chronic toxicity tests, reproduction assay, number of juveniles, based on ISO, 2014.

^h^ Earthworm (*Eisenia fetida*). Chronic toxicity test, reproduction assay, number of cocoons, based on ISO, 2012d.

Median effective concentration values are based on added metal concentrations. Except for data for wheat shoot (Smolders et al., 2003), data were collected from the *Threshold Calculator* database (Oorts, 2020).

PNR = potential nitrification rate; SIR = substrate‐induced respiration; MRM = maize residue mineralization.

The soils included in the study covered a wide range of soil properties. In the calibration soils, the pH value ranged from 3 to 7.5, the content of organic carbon varied between 0.4% and 23%, and the CEC varied between 2 and 36 cmol_c_ kg^−1^ (Table 1).

To enable further validation of the proposed concept, the results were compared with data published by Hamels et al. (2014), comprising data from 0.001 CaCl_2_ extracts and toxicity tests for barley shoot growth for seven European Zn‐contaminated soils as well as their freshly spiked references. These soils covered a pH range from 4.8 to 7.6, an organic carbon content from 1% to 23%, and a CEC from 1 to 69 cmol_c_ kg^−1^.

The available toxicity data include microbial tests, plant tests, and invertebrate tests. In plant and invertebrate tests, added species were exposed to the freshly spiked or field‐contaminated soils, whereas native populations were used in tests with microorganisms. *Freshly spiked* implies that the time between spiking and the start of the test was only 7 days. The toxicity tests lasted 4–28 days depending on the endpoint. Available data for spiked soils (1–22) are summarized in Table 2. These data were retrieved from the *Threshold Calculator* database (Oorts, 2020) and include fitted values for EC50, EC10, and the slope of dose–response curve (*S*, see Equation 1) obtained with nonlinear regression and a log‐logistic response curve in the program TRAP, Ver 1‐30a (US Environmental Protection Agency, 2015). Toxicity tests on the Cu‐contaminated field transects were performed in the studies of Oorts, Bronckaers, & Smolders (2006), Rooney et al. (2004), Criel et al. (2005; soil 23), and Ruyters et al. (2013; soils 25 and 26). Toxicity tests on the Zn‐contaminated field transect (soil 24) were performed in Smolders et al. (2003) and Lock et al. (2003).

### Spiking and batch leaching tests

To link the batch test data to the original toxicity data, the same sample pretreatment procedure was applied in the present study as in the original toxicity studies. All soil samples were preincubated for 1 week at 20 °C at a moisture content equivalent to 70% of field capacity (pF 2.0) before spiking. Uncontaminated soils were then spiked to 10, 30, 100, 300, 1000, and 3000 mg Cu  or Zn kg^−1^ dry soil, with CuCl_2_ or ZnCl_2_. Two soils were spiked with only five different concentrations because of limited amounts of available soil material, and two soils were spiked with an additional dose of 6000 mg kg^−1^ dry weight (Supporting Information, Table S1). In total, 21 soils were spiked with Cu and 17 soils with Zn. Finally, deionized water was added to the spike solution to adjust the soil moisture content to pF 2.0. Spiked soils were subsequently equilibrated for 1 week at 20 °C before the batch test. Spike solutions were analyzed to confirm the added doses.

Both freshly spiked and field‐contaminated soil samples were equilibrated with 0.001 M CaCl_2_ at a liquid‐to‐solid ratio of 10 according to ISO 21268‐2 (ISO, 2007). Samples (5 g dry wt with 50 ml solution) were equilibrated for 24 ± 0.5 h in acid‐washed polycarbonate vials at 10 rpm in an end‐over‐end shaker and then centrifuged at 4000 *g* for 15 min. The pH was measured on a portion of the eluate, and the rest was filtered through a 0.45‐µm filter before analysis of Cu, Zn, Ca, Na, K, Mg, Fe, Al, and dissolved organic carbon (DOC). Samples for elemental analysis were acidified with 5 µL ml^−1^ suprapure HNO_3_ before analysis with inductively coupled plasma (ICP) sector field mass spectrometry (Element1; Thermo Fisher) or ICP atomic emission spectroscopy (ICP optical emission spectrometer 725; Agilent). The DOC was determined by combustion and infrared detection (Nicolet Fourier transform infrared; Thermo Fisher) after acidification and removal of inorganic carbon. Chemical analyses were performed at laboratories accredited in accordance with the international standard ISO/IEC 17025 (ISO, 2005).

### Toxicity calculations

The toxic responses at different metal concentrations can be calculated from EC50 values and the slope of the dose–response curve (*S*) with the following equation:

(1) $$Y=\frac{Y_{0}}{1+e^{4S(\log X-\log\text{EC}50)}}$$

In Equation 1, *Y* is the effect relative to the reference at concentration *X*, *Y*_0_ is the response in the reference (no chemical exposure, here set to 1, i.e., no effect or 100% survival), and *X* is the metal concentration in soil (milligrams per kilogram dry wt) or batch test (milligrams per liter).

The EC*x* values for Cu and Zn, expressed as milligrams per kilogram dry soil, were translated to EC*x* expressed as milligrams per liter in soil extracts obtained in the batch test by a stepwise process. (1) Responses (*Y*) to added doses of Cu^2+^ or Zn^2+^ (milligrams per kilogram dry wt) for each toxicity test and soil were calculated with EC50 (milligrams per kilogram dry wt) and *S* values from the *Threshold Calculator* database (Oorts, 2020) using Equation 1. (2) Responses (*Y*) were paired with measured batch test concentrations of Cu and Zn at corresponding added doses (milligrams per kilogram dry wt), and a dose–response curve based on batch test concentrations was obtained for each toxicity test and soil by fitting the data with the log‐logistic nonlinear regression in TRAP, Ver 1‐30a. In this way, new EC10, EC50, and *S* values were obtained, based on concentrations in soil extracts obtained with batch tests (milligrams per liter) instead of added doses (milligrams per kilogram dry wt). Because “added Cu and Zn” was used as input, the concentration in the extract of the unspiked sample was subtracted from concentrations in extracts of spiked samples of the same soil to account for background concentrations of Cu or Zn. (3) The pH values in batch tests at EC50 (milligrams per liter) and EC10 (milligrams per liter) were interpolated from batch test data (pH as a function of Cu or Zn concentration in the leaching test) by a second‐degree polynomial function. This calculation was done in R, Ver 4.0.3 (R Foundation for Statistical Computing, 2020). Calculations according to these steps were made both for total Cu and Zn and for Cu^2+^ and Zn^2+^ concentrations in soil extracts. The corresponding calculations were performed for the field‐contaminated soil transects. To comply with added Cu and Zn doses, the background concentration of Cu or Zn (reference soil concentration) was first subtracted for field‐contaminated samples.

### Speciation calculations

The Cu and Zn speciation in the extracts from batch tests was calculated by Visual MINTEQ (Gustafsson, 2020). The following parameters were used as input data: (1) pH, DOC, and metal concentrations measured in extracts (metal concentrations were added in Visual MINTEQ as Cu^2+^, Zn^2+^, Ca^2+^, K^+^, Mg^2+^, Na^+^, Al^3+^, and Fe^3+^), and (2) Cl^−^ concentrations calculated from amounts added with spiking (CuCl_2_ or ZnCl_2_) and CaCl_2_ solutions. The Stockholm humic model (Gustafsson, 2001; Gustafsson & Van Schaik, 2003) was used with default parametrization for complexation with dissolved organic matter (DOM); the ratio of active DOM to DOC was 1.65, and all active DOM was assumed to be fulvic acid. We allowed Fe^3+^ and Al^3+^ to precipitate if the ion activity product exceeded the saturation index of ferrihydrite or aluminum hydroxide (Fh aged, log *K_s_ = 3.0 or Al[OH]_3_ soil, log *K_s_ = 8.6 at 20 °C [Linde et al., 2007]) because Fe and Al colloids (<0.45 µm) may overestimate the dissolved Fe and/or Al concentrations in 0.001 M CaCl_2_ leaching tests (Löv et al., 2018).

## RESULTS

### Cu and Zn in soil extracts of calibration soils

**Figure 2:**
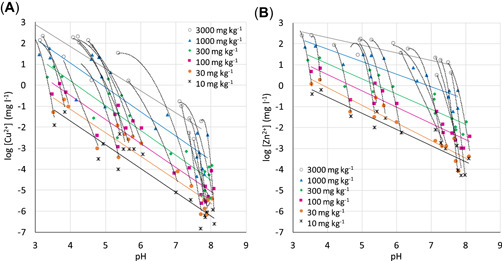
Concentrations of Cu^2+^ and Zn^2+^ as a function of pH in soil extracts: (**A**) [Cu^2+^] and (**B**) [Zn^2+^]. Symbols are concentrations in solution; solid lines are linear regressions for each added concentration over all soils. Dashed lines are regressions (two‐grade polynomial functions) for each soil over the range of added concentrations.

Extracts from batch tests conducted on the calibration soils covered a large range of dissolved Cu^2+^ and Zn^2+^ concentrations (Figure 2). At fixed added metal doses, Cu^2+^ and Zn^2+^ concentrations in extracts from the different soils decreased with increasing pH (Figure 2). The total Cu concentration in soil extracts ranged from approximately 1.5 to 40 µg l^−1^ at zero Cu^2+^ addition and from 160 to 200,000 µg l^−1^ at 3000 mg kg^−1^ added Cu^2+^. Total Zn concentrations ranged from approximately 100 µg l^−1^ with no Zn^2+^ added to between 5000 and 250,000 µg l^−1^ at 3000 mg kg^−1^ added Zn^2+^. The variation of pH (~pH 3–8; Figure 2) in the soil extracts could largely explain the difference in Cu^2+^ and Zn^2+^ at a certain added dose (*R* ^2^ = 0.84–0.91 for Cu^2+^ and 0.72–0.90 for Zn^2+^; Supporting Information, Table S2), despite a wide range in soil properties (Table 1). Thus, the pH of the soil extracts alone was a good predictor of dissolved Cu^2+^ and Zn^2+^ at a certain added dose.

As shown by the dashed black lines binding together results for the same soil in Figure 2, spiking lowered the pH. Two processes induced by the addition of metal chloride contribute to the pH decrease: (1) the increased ionic strength increases proton dissociation on soil organic matter (SOM), and (2) the added metals react with undissociated acidic SOM groups, resulting in proton release (Gustafsson & Kleja, 2005). The Cu^2+^ concentrations and pH in soil solutions from seven of the soils used in the present study have been measured by Criel et al. (2005; soils 2, 7, 10, 12, 18, 19, and 21). In their experiment, the soil solution was obtained by a centrifugation technique, and the Cu^2+^ concentration was measured by an ion‐selective electrode. The spiking procedure was the same as in the present study. A comparison reveals that pH in the soil solutions was lower and the Cu^2+^ concentration higher compared to the concentrations measured in extracts obtained with the batch test. Because of a lower liquid‐to‐solid ratio, this “spiking effect” is more pronounced in soil solutions than in soil extracts. However, the pH dependence of the Cu^2+^ concentration was very similar (Supporting Information, Figure S1). Consequently, the pH‐normalized Cu^2+^ concentrations in soil solution and soil extract from the same soil are similar, indicating that batch tests conducted with 0.001 CaCl_2_ solution can be used as a proxy for soil solutions. The corresponding comparison could not be made for Zn because of a lack of data on Zn^2+^ concentrations in the soil solutions.

### Toxic effects in spiked (calibration) soils and their pH dependence

**Figure 3:**
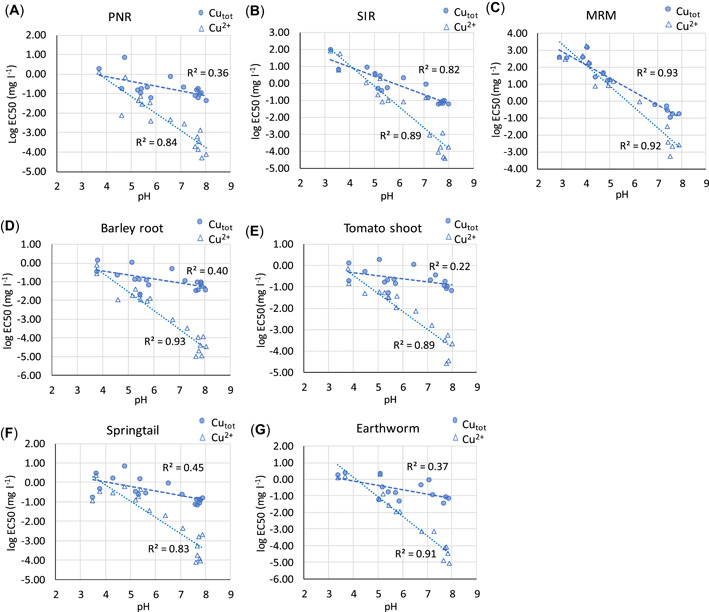
Calculated median effective concentration values based on [Cu_tot_] and [Cu^2+^] as a function of pH: (**A**) potential nitrification rate, (**B**) substrate‐induced respiration, (**C**) maize residue mineralization, (**D**) barley root elongation, (**E**) tomato shoot yield, (**F**) springtail reproduction, and (**G**) earthworm reproduction. PNR = potential nitrification rate; SIR = substrate‐induced respiration; MRM = maize residue mineralization; EC50 = median effective concentration.

**Figure 4:**
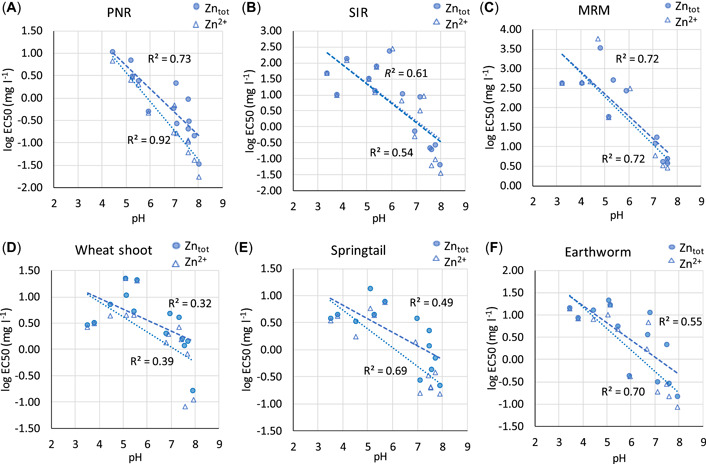
Calculated median effective concentration values based on [Zn_tot_] and [Zn^2+^] as a function of pH: (**A**) potential nitrification rate, (**B**) substrate‐induced respiration, (**C**) maize residue mineralization, (**D**) wheat shoot yield, (**E**) springtail reproduction, and (**F**) earthworm reproduction. PNR = potential nitrification rate; SIR = substrate‐induced respiration; MRM = maize residue mineralization; EC50 = median effective concentration.

Calculated EC50 values based on total Cu and Zn as well as Cu^2+^ and Zn^2+^ in soil extracts decreased with increasing pH, but the effect was smaller for Zn than Cu (Figures 3 and 4). The EC50s based on dissolved Cu^2+^ were strongly correlated to pH (*R* ^2^ ≥ 0.8 for all toxicity tests; Figure 3). The EC50s for total Cu concentration in soil extracts also correlated strongly with pH for substrate‐induced respiration (SIR) and maize residue mineralization (MRM), but the correlation was poor for the other tests, especially the tomato shoot test. The Zn EC50s correlated with pH; but the correlations were generally weaker than for Cu, and the difference between EC50s based on total Zn and Zn^2+^ in soil extracts was smaller, especially for SIR and MRM where regression lines overlap. This is due to the lower affinity of Zn for DOM, which leaves Zn in solution largely present as Zn^2+^.

The EC10 based on Cu^2+^ in soil extracts correlated with pH (*R* ^2^ > 0.7 for all regressions), although the correlation was weaker than that for EC50 (Table 3; Supporting Information, Figure S3). The pH dependence of both EC50 and EC10 based on Cu^2+^ in extracts was highly significant (*p* < 0.001) in all tests. The significance of the log EC*x*–pH correlations was often lower for Zn^2+^, especially for wheat shoot, which also had a low *R* ^2^ value (*R* ^2^ = 0.31). Similarly, Smolders et al. (2009), studying the same soils, demonstrated a larger statistical uncertainty for EC10 than EC50 based on metal concentrations in soil, which is reasonable because a smaller effect is more difficult to measure. In line with Smolders et al. (2009), it could be shown that the slopes of the EC50 and EC10 regressions were not significantly different for the same toxicity test, except for MRM for Cu^2+^. The *p* values from comparison of the slopes were >0.05 (MRM for Cu^2+^ *p* = 0.01; Supporting Information, Table S3).

**Table 3:** *R* ^2^ values of linear regressions for EC50 and EC10 values versus pH

Test^a^	Metal	*R* ^2^ EC50	*R* ^2^ EC10	Equation EC50 regression
PNR	Cu^2+^	0.84***	0.74***	Log EC50 = (−0.88 × pH) + 3.24
SIR		0.89***	0.89***	Log EC50 = (−1.22 × pH) + 5.97
MRM		0.92***	0.89***	Log EC50 = (−1.24 × pH) + 7.07
Barley root		0.93***	0.89***	Log EC50 = (−1.00 × pH) + 3.47
Tomato shoot		0.89***	0.82***	Log EC50 = (−0.84 × pH) + 2.92
Springtail		0.83***	0.76***	Log EC50 = (−1.18 × pH) + 4.85
Earthworm		0.91***	0.93***	Log EC50 = (−0.88 × pH) + 3.24
PNR	Zn^2+^	0.92***	0.82***	Log EC50 = (−0.65 × pH) + 3.82
SIR		0.54**	0.68***	Log EC50 = (−0.60 × pH) + 4.35
MRM		0.72**	0.41*	Log EC50 = (−0.61 × pH + 5.32
Wheat shoot		0.39*	0.30*	Log EC50 = (−0.29 × pH) + 2.06
Springtail		0.69***	0.61**	Log EC50 = (−0.35 × pH) + 2.14
Earthworm		0.70***	0.58**	Log EC50 = (−0.48 × pH) + 3.09

^a^ References are given in Table 2.

Regression equations were used to calculate pH‐dependent EC50 values.

EC50/EC10 = 50% and 10% effective concentrations; PNR = potential nitrification rate; SIR = substrate‐induced respiration; MRM = maize residue mineralization.

*p < 0.05, **p < 0.01, ***p < 0.001.

**Figure 5:**
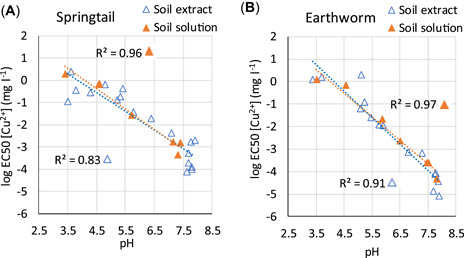
The pH dependence of median effective concentration (EC50) values for soil extracts (present study) and soil solutions (Criel et al., 2005): (**A**) EC50s for springtail based on measured [Cu^2+^] in soil solution and on [Cu^2+^] in soil extract (calculated), (**B**) EC50s for earthworm based on measured [Cu^2+^] in soil solution and [Cu^2+^] in soil extract (calculated).

As indicated, the pH‐dependent Cu^2+^ concentrations were similar in soil extracts and soil solution (Supporting Information, Figure S1). In addition to Cu^2+^ concentration data for soil solutions, Criel et al. (2005) provided EC50 values for springtail and earthworm based on Cu^2+^ concentrations for seven soils (2, 7, 10, 12, 18, 19, and 21 in Table 1). The EC50 values based on the measured Cu^2+^ concentration in soil solution had the same pH dependence as the EC50s based on the calculated Cu^2+^ concentrations in soil extracts (Figure 5). Thus, toxicity assessment based on the composition in the soil extracts will be the same as that based on the soil solution composition.

**Figure 6:**
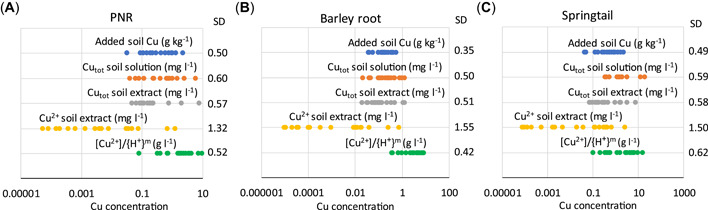
Comparison of five different expressions of median effective concentration (EC50) for Cu in 19 Cu‐spiked soils. The standard deviation of the log EC50 for each expression and test is given to the right in the graphs: (**A**) potential nitrification rate (PNR), (**B**) barley root elongation, (**C**) springtail reproduction. Soil solution data for PNR, barley root elongation, and springtail reproduction are from Oorts, Ghesquiere, et al. (2006), Zhao et al. (2006), and Criel et al. (2005), respectively. SD = standard deviation.

The EC50 based on pH‐dependent Cu^2+^ or Zn^2+^ concentrations in soil extracts (BLM approach) was compared with four other expressions of EC50 (Figure 6; Supporting Information, Figures S5 and S6). The pH‐normalized EC50 was calculated from the pH–EC50 relationships as EC50_free ion_/{H^+^}^m^, where m is the slope of the log EC50–pH relationship (Table 3). The other EC50 expressions were based on Cu and Zn added to soil (milligrams per kilogram dry wt), total Cu or Zn concentrations in soil solution, total Cu or Zn concentrations in soil extracts, and free ion concentrations in soil extracts.

The standard deviations (SDs) of log EC50 values based on pH‐normalized [Cu^2+^] or [Zn^2+^] were similar to those expressed as added concentration to soil (milligrams per kilogram dry wt), while the SDs of the other expressions of toxicity were higher (with a few exceptions). Notably, the concentration of free ions in soil extracts yielded the highest SDs. This clearly shows that competition effects by H^+^ ions need to be accounted for, in accordance with the BLM approach. The data in Figure 6 show that the average deviation from a “generic” EC50 value will be similar for the pH‐normalized (simplified BLM) EC50 as for EC50 for added concentration. It may be surprising that these added concentrations in different soils often show a smaller variation than the other expressions of bioavailability. The explanation for a relatively low SD of EC50 based on added concentrations, despite the large pH dependency of metal solubility, is the pH dependence of sorption to the biotic ligand, where high H^+^ concentrations counteract the toxicity of high metal ion concentrations at low pH as a result of competition (Smolders et al., 2009). However, the comparison in Figure 6 does not reveal how well the EC50 values would predict toxicity in field‐contaminated soils.

### Comparison of toxicity between spiked and field‐contaminated soils

**Figure 7:**
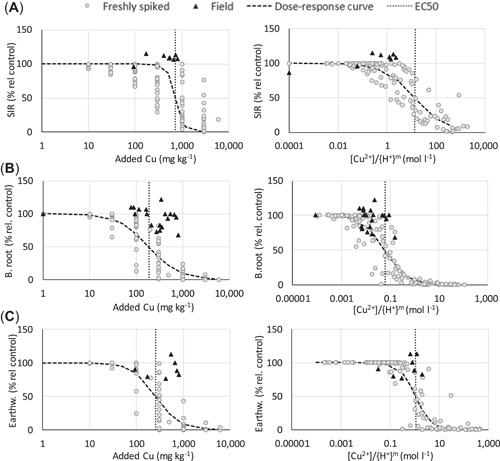
Responses in toxicity tests with Cu‐contaminated soils expressed as added concentration in soil (left) and as [Cu^2+^]/{H^+^}^m^ (right). Gray circles are data from all spiked soils; black triangles are available data for field‐contaminated soil transects (for barley root elongation from soils 23f, 25f, and 26f but for substrate‐induced respiration and earthworm reproduction only 23f). Dose–response curves were calculated for the spiked soils, and dashed vertical lines indicate the median effective concentration values. (**A**) Substrate‐induced respiration, (**B**) barely root elongation, and (**C**) earthworm reproduction. EC50 = median effective concentration; SIR = substrate‐induced respiration; B. = barley; Earthw. = earthworm.

**Figure 8:**
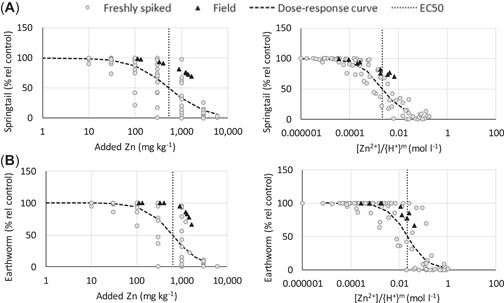
Responses in toxicity tests with Zn‐contaminated soils expressed as added concentration in soil (left) and as [Zn^2+^]/{H^+^}^m^ (right). Gray circles are data from all spiked soils; black triangles are data for field‐contaminated soil transect 24f. Dose–response curves were calculated for the spiked soils, and dashed vertical lines indicate the median effective concentration values. (**A**) Springtail and (**B**) earthworm reproduction. EC50 = median effective concentration.

The Cu^2+^ and Zn^2+^ concentrations in leachates of field‐contaminated soils and corresponding freshly spiked soils were similar at similar pH values (Supporting Information, Figure S2). The dose–response curves for toxicity tests based on added Cu^2+^ or Zn^2+^ concentrations in soil or pH‐normalized Cu^2+^ or Zn^2+^ concentrations in leachates (simplified BLM approach) were compared per test. A selection of dose–response curves is presented in Figures 7 and 8, and the remaining plots are available in Supporting Information, Figures S7 and S8.

When doses are expressed as the added soil concentration (milligrams per kilogram dry wt), the toxic effects at the same concentration of Cu and Zn were much larger in freshly spiked than field‐contaminated soils (Figures 7 and 8, left panels). Often, no toxic effect was identified in field soils even when the metal concentration was well above the EC50 values obtained for the spiked soils. However, the responses in toxicity tests with spiked and field‐contaminated soils converge when the free ion concentrations in the extracts are normalized for {H^+^} (Figures 7 and 8, right panels), indicating that the BLM approach gives a better estimate of toxicity in field‐contaminated soils.

**Figure 9:**
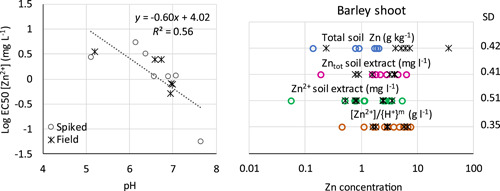
Log median effective concentration (EC50) values based on [Zn^2+^] for barley shoot test on Zn‐spiked and field‐contaminated soils and linear regression for spiked soils (left). Comparison of variation in different expressions of the log EC50 value (right); circles are spiked soils and 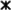 are field‐contaminated transects. The standard deviations calculated for spiked and field‐contaminated samples are given to the right. Based on data from Hamels et al. (2014). SD = standard deviation.

An additional validation of the simplified BLM approach was made based on data from Hamels et al. (2014). In the present study, EC50 values for barley shoot based on [Zn^2+^] in leachates were calculated for seven field‐contaminated soils and the corresponding spiked ones. The EC50 values for spiked and field‐contaminated soils showed a similar pH dependency when expressed as [Zn^2+^] (Figure 9). The SD for different expressions of the log EC50 values is shown in Figure 9B. Both spiked and field‐contaminated soils are included. The SD was lower for pH‐normalized concentrations of free ions in leachates compared to the other measures of toxicity. This confirms that the BLM approach improves the estimation of toxicity to soil organisms when toxicity data based on spiked soils are used to assess field‐contaminated soils.

## DISCUSSION

The present study corroborated earlier evidence that metal toxicity in soils is not explained by the free metal ion concentrations themselves. In contrast, toxic free metal ion concentrations vary largely among soils because of differences in pH (Figure 5; Supporting Information, Figures S5 and S6). This illustrates that the assessment of Cu and Zn toxicity in soils based directly on Cu^2+^ or Zn^2+^ in soil extracts may give misleading results, just as shown earlier for {Cu^2+^} in soil solutions (Smolders et al., 2009). To reflect the actual exposure of soil organisms to metals, the solution chemistry needs to be considered. Our data suggest that H^+^ is the key competing ion with Cu^2+^ and Zn^2+^ on the biotic ligand. The toxic effects (log EC50 and log EC10) based on Cu^2+^ or Zn^2+^ in soil extracts could largely be explained by hydrogen ion activity, although the regressions explain less of the Zn^2+^ toxicity (Table 3). To some extent, a contribution from other cations (e.g., Ca^2+^) may be indirectly included because their concentration in solution is positively correlated with pH. However, the overall effect on toxicity can be explained by pH. This is in accordance with the critical limit functions for Cu and Zn in soil (Lofts et al., 2004) that were derived based on {Cu^2+^} and {Zn^2+^}–pH relations as well as with the aquatic BLMs developed by De Schamphelaere et al. (2003, 2005). The previously developed TBLM model for Cu and Ni (Thakali et al., 2006a; Thakali et al., 2006b) included also competition with Ca^2+^ and Mg^2+^ on the biotic ligand, but for Cu, this competition was insignificant compared to protons. The slopes of the EC50–pH regressions (Table 3) are in line with previous studies. The Cu^2+^ slopes range between −0.88 (potential nitrification rate [PNR]) and −1.24 (MRM), with an average of −1.03, which is close to the results from Lofts et al. (2013) where slopes were between −0.63 (PNR) and −1.15 (SIR) and the average was −0.95. Our slopes for Zn^2+^ regressions were between −0.29 (wheat shoot) and −0.65 (PNR) with an average of −0.5. We did not find any similar studies for soils, but De Schamphelaere et al. (2005) calculated a slope of −0.65 for a regression including three freshwater organisms (*Pseudokirchneriella subcapitata*, *Daphnia magna*, and *Oncorhynchus mykiss*).

Unlike earlier BLM approaches for soils, the current model was developed for evaluation of a standardized leaching test (batch test). By this approach we avoid the uncertainties introduced by a step where the soil–solution partitioning of metals is predicted using a geochemical model (as in the TBLM by Thakali et al., 2006a; Thakali et al., 2006b). Ideally, a BLM approach could be applied to the soil solutions, as in the work of Lofts et al. (2013); but soil solution samples are laborious to obtain in sufficient quantities. In contrast, standardized leaching tests are routinely performed at commercial laboratories and already commonly used in risk assessments of contaminated sites for assessment of contaminant mobility. Leaching tests based on dilute CaCl_2_ (e.g., 0.01 or 0.001 M CaCl_2_) can be used as tools to predict metal concentrations in soil solutions of field soils (Degryse et al., 2003). Our study implies that toxicity assessments based on a 0.001 M CaCl_2_ batch test will be the same as those based on soil solutions for freshly spiked soils if they are combined with a BLM approach. Although soil solutions from freshly spiked soils generally contain higher concentrations of metals and have lower pH than the corresponding soil extracts, the toxic effect (EC50) on springtail and earthworm was the same for soil solution and soil extract if expressed as pH‐normalized [Cu^2+^] (i.e., the same slope; Figure 5). Similarly, the pH‐normalized Cu^2+^ concentration in the soil extract corresponded to the pH‐normalized Cu^2+^ concentration in the soil solution at the same added Cu concentration (Supporting Information, Figure S1). Thus, the toxicity effect caused by the higher Cu^2+^ concentrations in soil solutions compared to soil extracts is being counteracted by the higher hydrogen ion concentration, resulting in the same pH‐dependent toxicity of Cu^2+^ in soil solutions and in the 0.001 M CaCl_2_ leaching test.

Other similar models have successfully used a universal slope for the pH‐dependent toxicity of several different species (De Schamphelaere & Janssen, 2006; Lofts et al., 2004). A generic slope for our EC*x*–pH relationships would be beneficial to simplify the BLM and to include more test organisms/endpoints in the development of EQS/guideline values. Environmental quality standards are derived from EC10, predicted‐no‐effect concentration, or NOEC values. Currently, our model is based on the more stable EC50 values, but similar to Smolders et al. (2009), we showed that the slopes of the EC50 and EC10 regressions for the same toxicity test were not statistically different, with the exception of the MRM test (*p* values in Supporting Information, Table S3). The slopes (m) of the EC50 regressions for different toxicity tests with the same metal are rather similar (differing by a maximum of 0.4; Supporting Information, Table S3). Comparing the EC10 regressions, the MRM test for Cu^2+^ stands out with a steeper slope than the other tests (Supporting Information, Table S3). The explanation is a flat dose–response curve (Supporting Information, Figures S7 and S8), resulting in an uncertain estimate of the EC*x* values. Accordingly, our data support the use of a generic slope for most organisms.

In the approach taken, we would automatically include aging effects and solid‐phase metal speciation in the evaluation. As a result, data from toxicity experiments made with spiked soils can be used during calibration, without any corrections. As shown in this and other studies (Oorts, Bronckaers, & Smolders, 2006; Smolders et al., 2004), the toxic effect based on added or total soil concentration (milligrams per kilogram dry wt) is larger in freshly spiked soils compared to field‐contaminated soils with similar soil properties. This is due to lower solution concentrations in field‐contaminated soils at a certain soil metal concentration. In our study there was often no detectable toxic effect in field soils even when the metal concentration in the field soil was above the EC50 value for the freshly spiked soil (i.e., Figures 7 and 8, left panels). The toxicity assessment made using the simplified BLM showed a much closer agreement between field‐contaminated soils and freshly spiked soils (Figures 7 and 8, right panels). Accordingly, neither normalization to soil properties nor correction using laboratory‐field factors are needed using this approach.

In the work of Hamels et al. (2014), a range of soil tests for diagnosing phytotoxicity in contaminated soils were investigated, including 0.43 M HNO_3_, 0.05 M ethylenediaminetetraacetic acid, 1 M NH_4_NO_3_, cobaltihexamine, diffusive gradients in thin films, and 0.001 M CaCl_2_. Both spiked and field‐contaminated soils were included and the evaluation showed that the 0.001 M CaCl_2_ batch test was the most robust test for Zn phytotoxicity among all the methods investigated. The data of Hamels et al. (2014) were further evaluated in the present study, using the BLM approach. As shown in Figure 9, the variation between soils, including both spiked and field‐contaminated, decreased further when applying the BLM approach to soil extracts obtained with the batch test compared to using total soluble Zn as a toxicity measure. This is in line with the significant correlation between EC50 and pH in soil extracts (Figure 9). In addition, the agreement between spiked and field‐contaminated soils was much better using the BLM approach. This can be seen by comparing the circles (spiked soils) with 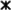 (field‐contaminated soils) on the same row (as well as in Figures 7 and 8).

## IMPLICATIONS

Assessing ecotoxicity of Cu and Zn in soils using a standard batch test in combination with a simplified BLM approach could provide a useful tool to improve site‐specific ecotoxicological risk assessments. Current ISO standards mention leaching tests as possible tools to assess bioavailability (ISO, 2008a, 2017) but do not give any guidance regarding calibration of models or evaluation of results. The proposed concept can be used as a complement to established methods for ecological risk assessments of metals in soils, for example, the *Threshold Calculator*. The *Threshold Calculator* is a flexible tool for different levels (tiers) of risk assessment covering several metals. However, one advantage with the BLM approach in site‐specific risk assessment is that no site‐specific laboratory‐field factor needs to be determined because “spiking” and “aging” effects are already accounted for in the BLM approach. In addition, the correction functions in the *Threshold Calculator* were developed on assemblages of soil components in natural soils. Urban soils may contain other sorbents like ash, soot, and amendment with biochars, with properties that differ from natural soil components, making the *Threshold Calculator* less appropriate in such cases. Another advantage is that the same leaching test can be used for assessment of the mobility of metals in soils, e.g., the risk for further transport to groundwater and surface water.

The work presented in our study provides a first step in developing an easy‐to‐use procedure for derivation of site‐specific soil guideline values/EQS. These values are commonly based on hazardous concentrations (HCs) for protecting different numbers of species and/or biological processes, preferably derived from species sensitivity distribution (SSD) functions. The use of such a procedure in practice would require extraction of field‐contaminated soil samples with CaCl_2_ following ISO 21268‐2 (ISO, 2007) and chemical analysis of the leachate including metals, main cations, pH, and total organic carbon. The calculated free metal concentration should then be compared with pH‐corrected HC*x* values derived from BLM‐based SSD functions. To derive the SSD functions, the current toxicity data sets need to be complemented by more species/endpoints. According to the guidance document from the European Chemicals Agency (2008), an SSD should comprise data for at least 10, and preferably more than 15, species from several taxonomic groups to derive HC*x* values for surface waters. The present data set for Cu contains data for seven species (three microbial, two plants, and two invertebrates). If the data set from Hamels et al. (2014) is included, Zn data also comprise seven species (three microbial, two plants, and two invertebrates). Consequently, data for more species are need to be collected to fulfill the criteria of a robust SSD. The regressions now derived (simplified BLMs) suggest that generic slopes can be used for Cu and Zn, which facilitates further method development.

## Supporting Information

The Supporting Information is available on the Wiley OnlineLibrary at https://doi.org/10.1002/etc.5326.

## Disclaimer

The authors have no known competing financial or other interests related to the present study.

## Author Contributions Statement

**Charlotta Tiberg:** Conceptualization; Data curation; Formal analysis; Methodology; Writing—original draft. **Erik Smolders:** Conceptualization; Resources; Methodology; Writing—review and editing. **Mats Fröberg:** Formal analysis; Writing—review and editing. **Jon Petter Gustafsson:** Conceptualization; Methodology; Writing—review and editing. **Dan Berggren Kleja:** Conceptualization; Formal analysis; Methodology; Writing—review and editing.

## Supporting information

This article contains online‐only Supporting Information.

Supporting information.

## Data Availability

Used and mentioned software/calculation tools are freeware. Links are given in the reference list. For toxicity data, EC50 and EC10 values based on Cu or Zn added to soil were collected from the database *Threshold Calculator of Metals in Soil* and complemented with data from the literature (all references are given in the reference list). These data were used to calculate effects at different concentrations in spiked soils. Data (complete data from batch tests, speciation calculations, as well as calculated EC50s and EC10s), associated metadata, and calculation tools are available from the corresponding author (charlotta.tiberg@sgi.se).
