# Supplementary material for: Combining a Standardized Batch Test with the Biotic Ligand Model to Predict Copper and Zinc Ecotoxicity in Soils
Source: Environ Toxicol Chem. 2022 Apr 18;41(6):1540–54. doi: 10.1002/etc.5326 (PMC9325525; doi:10.1002/etc.5326)
Supplement: Supplementary file 1 — Supporting information. [file ETC-41-1540-s001.docx]

**Supporting information**

*Table S1. Soils and added metal concentrations.*

| **Soil nr** | **Soil** | **Spike metal** | **Spiked concentration (mg/kg dw)** |
| --- | --- | --- | --- |
| 1 | Gudow | Cu, Zn | 0, 10, 30, 100, 300, 1000, 3000, 6000 |
| 2 | Nottingham | Cu | 0, 10, 30, 100, 300, 1000, 3000 |
| 3 | Houthalen | Cu, Zn | 0, 10, 30, 100, 300, 1000, 3000 |
| 4 | Rhydtalog | Cu, Zn | 0, 10, 30, 100, 300, 1000, 3000 |
| 5 | Zegveld | Cu, Zn | 0, 10, 30, 100, 300, 1000, 3000, 6000 |
| 6 | Rhydtalog c.t. | Zn | 0, 10, 30, 100, 300, 1000, 3000 |
| 7 | Kövlinge I | Cu | 0, 10, 30, 100, 300, 1000, 3000 |
| 8 | Souli I | Cu, Zn | 0, 10, 30, 100, 300, 1000, 3000 |
| 9 | Kövlinge II | Cu, Zn | 0, 10, 30, 100, 300, 1000, 3000 |
| 10 | Montpellier | Cu | 0, 10, 30, 100, 300, 1000, 3000 |
| 11 | De Meern | Zn | 0, 10, 30, 100, 300, 1000, 3000 |
| 12 | Aluminusa | Cu, Zn | 0, 10, 30, 100, 300, 1000, 3000 |
| 13 | Zeveren | Zn | 0, 10, 30, 100, 300, 1000, 3000 |
| 14 | Woburn | Cu, Zn | 0, 10, 30, 100, 300, 1000, 3000 |
| 15 | Ter Munck | Cu, Zn | 0, 10, 30, 100, 300, 1000, 3000 |
| 16 | Vault de Lugny | Cu | 0, 10, 30, 100, 300, 1000, 3000 |
| 17 | Rots | Cu, Zn | 0, 10, 30, 100, 300, 1000, 3000 |
| 18 | Souli II | Cu, Zn | 0, 10, 30, 100, 300, 1000, 3000 |
| 19 | Marknesse | Cu, Zn | 0, 10, 30, 100, 300, 1000, 3000 |
| 20 | Barcelona | Cu | 0, 10, 30, 100, 300, 1000, 3000 |
| 21 | Brécy | Cu | 0, 10, 30, 100, 300, 1000, 3000 |
| 22 | Guadalajara | Cu, Zn | 0, 10, 30, 100, 300, 1000, 3000 |
| 23c | Hygum control | Cu | 0, 10, 30, 100, 300, 1000, 3000 |
| 23f | Hygum field | Field Cu | Field contaminated |
| 24f | Zeveren, field | Field Zn | Field contaminated |
| 24c | Zeveren control | Zn | 0, 30, 300, 1000, 3000 |
| 25f | Navicello field | Field Cu | Field contaminated |
| 26c | Wincheringen control | Cu | 0, 30, 300, 1000, 3000 |
| 26f | Wincheringen field | Field Cu | Field contaminated |

*Table S2. The R^2^-values of linear regressions for the Cu^2+^ or Zn^2+^ concentrations and pH in batch tests at different added Cu and Zn (Figure 2, main text).*

| Added concentration | R-value Cu | R-value Zn |
| --- | --- | --- |
| 10 mg kg^-1^ | 0.90 | 0.72 |
| 30 mg kg^-1^ | 0.91 | 0.79 |
| 100 mg kg^-1^ | 0.88 | 0.85 |
| 300 mg kg^-1^ | 0.88 | 0.89 |
| 1000 mg kg^-1^ | 0.84 | 0.90 |
| 3000 mg kg^-1^ | 0.87 | 0.86 |

*Figure S1. Cu^2+^ concentrations in soil extract (calculated with Visual MINTEQ) and soil solution (measured with ion selective electrode) in soils spiked with a) 100 mg kg^-1^ Cu and b) 300 mg kg^-1^ Cu. The coloured lines are linear regressions and black lines tie together datapoints for soil extract and soil solution for the same soil.*

*Figure S2. Cu^2+^ and Zn^2+^ concentrations in soil extracts from spiked and field contaminated soils. Dependence on added/background corrected Cu and Zn. Letter s denotes spiked and f field contaminated soil samples.*

*Figure S3. Calculated EC50 and EC10 values for Cu^2+^ related to pH; a) Nitrification, b) Glucose induced respiration, c) Maize residue mineralization, d) Barley root elongation, e) Tomato shoot growth, f) Springtail reproduction and g) Earthworm reproduction.*

*Figure S4. Calculated EC50 and EC10 values for Zn^2+^ related to pH; a) Nitrification, b) Glucose induced respiration, c) Maize residue mineralization, d) Wheat shoot growth, e) Barley shoot growth, f) Springtail reproduction and g) Earthworm reproduction.*

*Table S3. Slopes (m) of logEC50-pH and logEC10-pH linear regressions (Figure S3 and S4) and p-values for comparison of slopes for EC50 and EC10 regressions.*

|  | m  Log EC50 Cu^2+^-pH | m  Log EC10 Cu^2+^-pH | m  Log EC50 Zn^2+^-pH | m  Log EC10 Zn^2+^-pH | p-values m Cu^2+^ EC50, EC10 | p-values m Zn^2+^ EC50, EC10 |
| --- | --- | --- | --- | --- | --- | --- |
| PNR | -0.88 | -1.00 | -0.65 | -0.69 | 0.50 | 0.79 |
| SIR | -1.22 | -1.41 | -0.60 | -0.69 | 0.26 | 0.68 |
| MRM | -1.24 | -1.76 | -0.61 | -0.52 | 0.01 | 0.73 |
| B. root | -1.00 | -1.09 | n.a. | n.a. | 0.45 | --- |
| T. shoot | -0.84 | -0.90 | n.a. | n.a. | 0.65 | --- |
| W. shoot | n.a. | n.a. | -0.29 | -0.36 | --- | 0.70 |
| Springtail | -0.84 | -0.81 | -0.35 | -0.46 | 0.83 | 0.41 |
| Earthworm | -1.18 | -1.21 | -0.48 | -0.66 | 0.96 | 0.36 |

*Figure S5. Comparison of five different expressions of EC50 for Cu in 19 Cu spiked soils. The standard deviation for each expression and test is given to the right in the graphs, a) Substrate induced respiration, b) Maize residue mineralization, c) Tomato shoot yield and, d) Earthworm reproduction.* *Soil solution data for SIR, tomato shoot elongation and earthworm are from Oorts et al (2006b), Zhao et al. (2006) and Criel et al. (2005) respectively.*

*Figure S6. Comparison of five different expressions of EC50 for Zn in Zn spiked soils. The standard deviation for each expression and test is given to the right in the graphs, a) Nitrification, b) Glucose induced respiration, c) Maize residue, d) Wheat shoot, e) Springtail reproduction and, f) Earthworm reproduction. Soil solution data from Smolders et al. (2003) and Lock et al. (2003).*

*Figure S7. Responses in toxicity tests with Cu*-*contaminated soils expressed as added concentration to soil (left row) and as [Cu^2+^]/{H^+^}^m^ (right row). Grey circles are data from spiked soils, black triangles are the field contaminated soil transects (23f, 25f and 26f for PNR and T. shoot, remaining only 23f). Dose-response curves were calculated for the spiked soils and dashed vertical lines indicate the EC50 values. a) Nitrification, b) Maize residue mineralization, c) Tomato shoot growth and d) Springtail reproduction.*

*Figure S8. Responses in toxicity tests with Zn contaminated soils expressed as added concentration to soil (left row) and as [Zn^2+^]/{H^+^}^m^ (right row). Grey circles are data from all spiked soils, black triangles are data for field contaminated soil transect 24f. Dose-response curves were calculated for the spiked soils and the dashed vertical lines indicate the EC50 values. a) Nitrification, b) Substrate induced respiration, c) Maize residue mineralization, d) Wheat shoot growth.*

## References

Criel, P., De Schamphelaere, K.A.C., Janssen. C. R. (2005). *Development of a predictive model of bioavailability and toxicity of copper in soils - Invertebrate toxicity. Final report for the International Copper Association*. Laboratory of Environmental Toxicology and Aquatic Ecology Ghent University, Gent, Belgium

Lock, K., Criel, P., Janssen, C.R. (2003). *Laboratory Zn Ecotoxicity testing for soil invertebrates (Final report of ILZRO project ZEH-SO-3),* Laboratory of Environmental Toxicology and Aquatic Ecology, Ghent University, Gent, Belgium.

Oorts, K., Ghesquiere, U., Swinnen, K., Smolders, E.. (2006b). Soil properties affecting the toxicity of CuCl2 and NiCl2 for soil microbial processes in freshly spiked soils. *Environ Toxicol Chem., 25*(3):836–844. <https://doi.org/10.1897/04-672R.1>

Smolders, E., Buekers, J., Waegeneers, N., Oliver I., McLaughlin M. (2003). *Effects of Field and Laboratory Zn Contamination on Soil Microbial Processes and Plant Growth (Final report of ILZRO project ZEH-SO-2)*, Laboratory of Soil and Water Management, Leuven University, Heverlee, Belgium, and CSIRO Land and Water, Adelaide, Australia.

Zhao, F.J., Rooney, C.P., Zhang, H., McGrath, S.P. (2006). Comparison of soil solution speciation and diffusive gradients in thin-films measurement as an indicator of copper bioavailability to plants. *Environ Toxicol Chem. 25*(3), 733–742. <https://doi.org/10.1897/04-603R.1>
